# Mapping stunted children in Ethiopia using two decades of data between 2000 and 2019. A geospatial analysis through the Bayesian approach

**DOI:** 10.1186/s41043-023-00412-3

**Published:** 2023-10-26

**Authors:** Kendalem Asmare Atalell, Masresha Asmare Techane, Bewuketu Terefe, Tadesse Tarik Tamir

**Affiliations:** 1 Department of Pediatrics and Child Health Nursing, School of Nursing, College of Medicine and Health Sciences, University of Gondar, Gondar, Ethiopia; 2 Department of Community Health Nursing, School of Nursing, College of Medicine and Health Sciences, University of Gondar, Gondar, Ethiopia

**Keywords:** Children, Geospatial, Stunting, Temporal, Under-five

## Abstract

**Background:**

Childhood stunting is a major public health problem globally, resulting in poor cognition and educational performance, low adult wages, low productivity, and an increased risk of nutrition-related chronic diseases in adulthood life. Accurate and reliable data on the prevalence of stunting over time with a sub-national estimate are scarce in Ethiopia.

**Objective:**

Our objective was to investigate the spatiotemporal distributions and ecological level drivers of stunting among under-five children over time in Ethiopia.

**Methods:**

A geospatial analysis using the Bayesian framework was employed to map the spatial variations of stunting among children aged less than five years. The data for the primary outcome were obtained from the Ethiopian Demographic and Health Surveys (2000–2019) and covariates data were accessed from different publicly available credible sources. The spatial binomial regression model was fitted to identify drivers of child stunting using the Bayesian approach.

**Result:**

The national prevalence of stunting was 47.9 in 2000, 43.3 in 2005, 37.3 in 2011, 36.6 in 2016, and 35.9 in 2019, with a total reduction rate of 25%. Substantial spatial clustering of stunting was observed in the Northern (Tigray), Northcentral (Amhara), and Northwestern (Amhara) parts of Ethiopia. Temperature (mean regression coefficient (β): −0.19; 95% credible interval (95% CrI): −0.25, −0.12) and population density (β: −0.012; 95% CrI: −0.016, −0.009) were negatively associated with stunting, whereas travel time to the nearest cities (β: 0.12; 95% CrI: 0.064, 0.17) was positively associated with child stunting in Ethiopia.

**Conclusion:**

The prevalence of stunting varied substantially at subnational and local levels over time. Clustering of stunted children were observed in the Northern parts of Ethiopia. Temperature, population density and travel time to the nearest cities were identified as the drivers of stunting in children. Improving community awareness of child nutrition through community health extension programs should be strengthened.

## Introduction

Stunting is the major public health concern in developing countries like Ethiopia, which is caused by chronic and recurrent malnutrition that leads to growth failure both physically, psychologically, and cognitively [1]. According to the World Health Organization (WHO), stunting is defined as length/height per age being less than -2 Standard Deviations (SD) [2]. Although childhood stunting is reduced in the past two to three decades, it is still a major public health problem globally, which resulted in poor cognition and educational performance, low adult wages, low productivity, and an increased risk of nutrition-related chronic diseases in adulthood life [1].

Globally, about 149 million under-five children are suffering from stunting with a defined geographical variation [3]. The magnitude in developing countries was 43.4% [4], and 90% of the global burden of stunting was attributed to 36 African and Asian countries [5]. Moreover, the Sab-Saharan African countries accounted for more than one-third of the world's stunted children [6]. Stunting is a major public health threat in Ethiopia, which affects more than Ethiopia 42% of under-five children [7], with significant geographical variations over time in Ethiopia [8, 9]. Previous studies conducted in Ethiopia tried to explore the spatial variations and individual level determinants of stunting in children [10–14]. But those studies lack to identify the population level climatic and demographic variables. Hence, understanding the spatial variations of stunting children over time and identifying the population level drivers would inform policymakers to come up with geographically targeted interventions.

Efforts have been made to reduce stunting by 40% in 2025 globally, and the Ethiopian government has planned to end child undernutrition specifically stunting by 2030 using the ‘Seqota’ declaration [15], which aimed to improve child nutrition through improving the agricultural activities, safety net programs including school feeding program [16]. However, the magnitude of stunting is still huge that pursues actions oriented to specific geographic regions. Thus, we aimed to investigate the spatiotemporal variations of stunting in Ethiopia and identify drivers of stunting over time to come up with an evidence-based intervention to reduce stunting in Ethiopia.

## Methods

### Ethiopian context

The study was conducted in Ethiopia, which is the third largest country in Africa with a total population size of 115 million. The country has varied altitudes ranging from 125 m below sea level in Afar to 4663 m above sea level in Ras Dashen, Amhara. Ethiopia is among the oldest countries in the world, which is the home of 13 [4 tangible and 9 intangible] UNESCO registered heritages such as the Rock-Hewn churches of Lalibela, Fasil Ghebbi, Konso Cultural landscapes, etc. Administratively, Ethiopia is classified as 11 regions and 2 chartered cities. More than 78% of the population in Ethiopia resides in rural. More than half of the population in Ethiopia travels more than 10 km to get to health facilities. Stunting is a major health problem in Ethiopia.

### Study participants

We conducted ecological study using the Ethiopian Demographic and Health Surveys conducted from 2000 to 2019.

Under-five children included in the five EDHSs in Ethiopia were included in this analysis. All the five EDHS enumeration areas were stratified into urban and rural following the nearby population and housing censuses. The EDHS used a two-stage stratified cluster sampling. In the first stage clusters/enumeration areas were selected using probability sampling. In the second stage, households in the selected cluster were selected using probability sampling. Mothers aged 15–49 years in each selected household were interviewed and anthropometry was taken for all under-five children in each household. Data for our analysis were extracted from the record of reproductive-age women and under-five children in each of the five EDHSs. The survey design was described elsewhere [17–21]. We have included a total of 36,013 under-five children in this analysis, 8590 in 2000, 3873 in 2005, 9619 in 2011, 8855 in 2016, and 5076 in 2019 in our analysis.

### Variables and data sources

The outcome variable for this study is stunting among under-five children, which was obtained from the EDHSs data between 2000 and 2019. A child is considered stunted if the height for age of the child becomes below -2 Standard Deviations (SD). The EDHS data is nationally representative data, which is conducted every five years between 2000 and 2019. There are five different EDHSs (i.e., 2000, 2005, 2011, 2016 and 2019). Geospatial covariate data were obtained from several sources with a resolution of 1 km^2^. Climatic data such as temperature and precipitation were accessed from the WorldClim website [22]. Distance to the nearest cities and access to healthcare facilities data were obtained from the Malaria Atlas Project (MAP) [23]. Population density and distance to waterbody data were retrieved from WorldPop [24] and Global Lakes and Wetlands Database (GLWD), respectively. The variables were selected based on the availability of high-resolution countrywide data. The polygon shapefile for the Ethiopian administrative boundaries was obtained from the Global Administrative Areas (GADEM) a free online database. The proportion of stunting was georeferenced and linked with area level covariates using ArcGIS.

### Data processing and analysis

The EDHS data were accessed at the MEASURE DHS website through formal registrations and requests. The Kids Records (KR) datasets were used for this analysis. Descriptive statistics such as the proportion of stunting in each administrative region were calculated and presented in the table and the trend of stunting through the last two decades was estimated and presented in the graph. The outcome variable was classified as stunting if the child is below −2SD.

### Spatial analysis

Geospatial analysis using the Bayesian approach was used to generate a spatially continuous estimate of the national proportion of stunting mapped in 2000, 2005, 2011, 2016, and 2019 EDHS surveys at a resolution of 1 km^2^. The binomial regression model was fitted within the Bayesian framework to the proportion of stunting of both fixed effects and geostatistical random effects. Five models were constructed separately for the proportion of stunting in 2000, 2005, 2011, 2016, and 2019 EDHS data. The model for the stunting was the same for all five datasets. A spatial binomial regression model was fitted for stunting survey data including fixed effects for temperature, precipitation, travel time to the nearest city, distance to the nearest health facilities, distance to the water body and population density, and geostatistical random effects [25]. The proportion of stunting was taken at each surveyed location *j* as the outcome variable, which was assumed to follow a binomial distribution:

$$Y_{j} \sim Binomial\left( {n_{j} ,p_{j} } \right);$$

where ${Y}_{j}$ are the observed stunted children, ${n}_{j}$ is the total number of children in each survey and ${p}_{j}$ is the predicted proportion of stunting at location $j$(j = 1, …535 for 2000 and 517 for 2005, 571 for 2011, 619 for 2016, and 305 for 2019 EDHSs). The mean predicted was the proportion of stunting was modeled via a logit link function to a linear predictor defined as:

$$\log it\left( {p_{j} } \right)\alpha + \sum\limits_{z = 1}^{z} {\beta_{z} X_{z,j} } + \zeta_{j} ;$$

where *α* is the intercept, *β* is a matrix of covariate coefficients, ${\varvec{X}}$ is a design matrix of $z$ covariates, and ${\zeta }_{j}$ are spatial random effects modeled using a zero-mean Gaussian Markov random field (GMRF) with a Matérn covariance function. The covariance function was defined by two parameters: the range $\rho$, which represents the distance beyond which correlation becomes negligible (about 0.1), and $\sigma$, which is the marginal standard deviation [26]. Non-informative priors were used for *α* (uniform prior with bounds –∞ and ∞), and we set normal priors with mean = 0 and precision (the inverse of the variance) = 1 × 10^–4^ for each *β*. We used default priors for the parameters of the spatial random field [27]. Parameter estimation was done using the Integrated Nested Laplace Approximation (INLA) approach in R (R-INLA) [26, 28]. Sufficient values (i.e., 150,000 samples) from each simulation run for the variables of interest were stored to ensure full characterization of the posterior distributions.

Predictions of stunting at unsampled locations were made at 1 km^2^ resolution by interpolating the spatial random effects and adding them to the sum of the products of the coefficients for the spatially variant fixed effects at each prediction location [29]. The intercept was added, and the overall sum was back transformed from the logit scale to the prevalence scale, providing prediction surfaces that show the estimated immunization coverage for all prediction locations. The covariate correlation matrix was checked, and altitude was removed because of its interaction with temperature.

The Watanabe Applicable Information Criterion (WAIC) statistic was used to select the best-fitting model.

## Result

### Geographical and temporal variations

We accessed a total of 37,969 under five children in the five EDHSs, of those 1956 were excluded due to 1220 flagged cases and 836 height/age not recorded. Thus, 36,013 under-five children were included in the analysis. The overall prevalence of stunting in Ethiopia was 47.9%, 43.3%, 37.3%, 36.6% and 35.9% in 2000, 2005, 2011, 2016 and 2019, respectively. The prevalence of stunting was higher in Amhara and Tigray regions in all EDHSs (Table 1).

**Table 1:** The national and regional prevalence of stunting among under-five children over time

Regions	Prevalence of stunting				
	2000	2005	2011	2016	2019
Tigray	55.8	42.5	45.9	39.7	51.5
Afar	49.4	40.4	45.9	43.9	43.5
Amhara	57.6	56.7	45.3	47.5	43.7
Oromia	47.4	41.4	35.6	36.3	37.1
Somali	48.5	46.3	28.8	27.6	31.0
Benishangul-gumz	41.9	40.9	43.8	44.3	41.0
SNNPR	53.9	50.2	39.6	38.7	37.8
Gambela	39.6	30.8	23.7	26.3	19.4
Harari	37.7	39.5	24.5	31.7	33.7
Addis Ababa	28.6	18.6	15.5	14.8	14.3
Dire Dawa	32.5	27.8	33.2	39.9	26.7
Ethiopia	47.9	43.3	37.3	36.6	35.9

*SNNPR* Southern Nations, Nationalities and Peoples Region

**Fig. 1:**
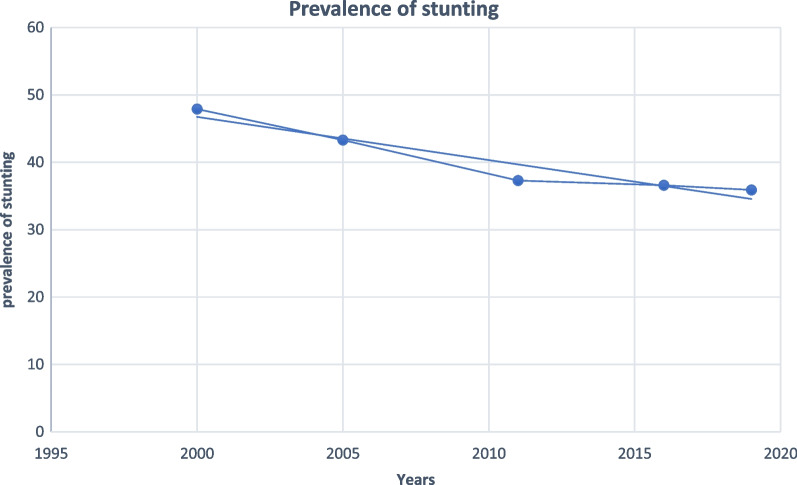
Trends of stunting among under-five children in Ethiopia between 2000 and 2019

The prevalence of stunting was reduced by 25% in the last two decades from 47.9% in 2000 to 35.9% in 2019 (Fig. 1).

**Fig. 2:**
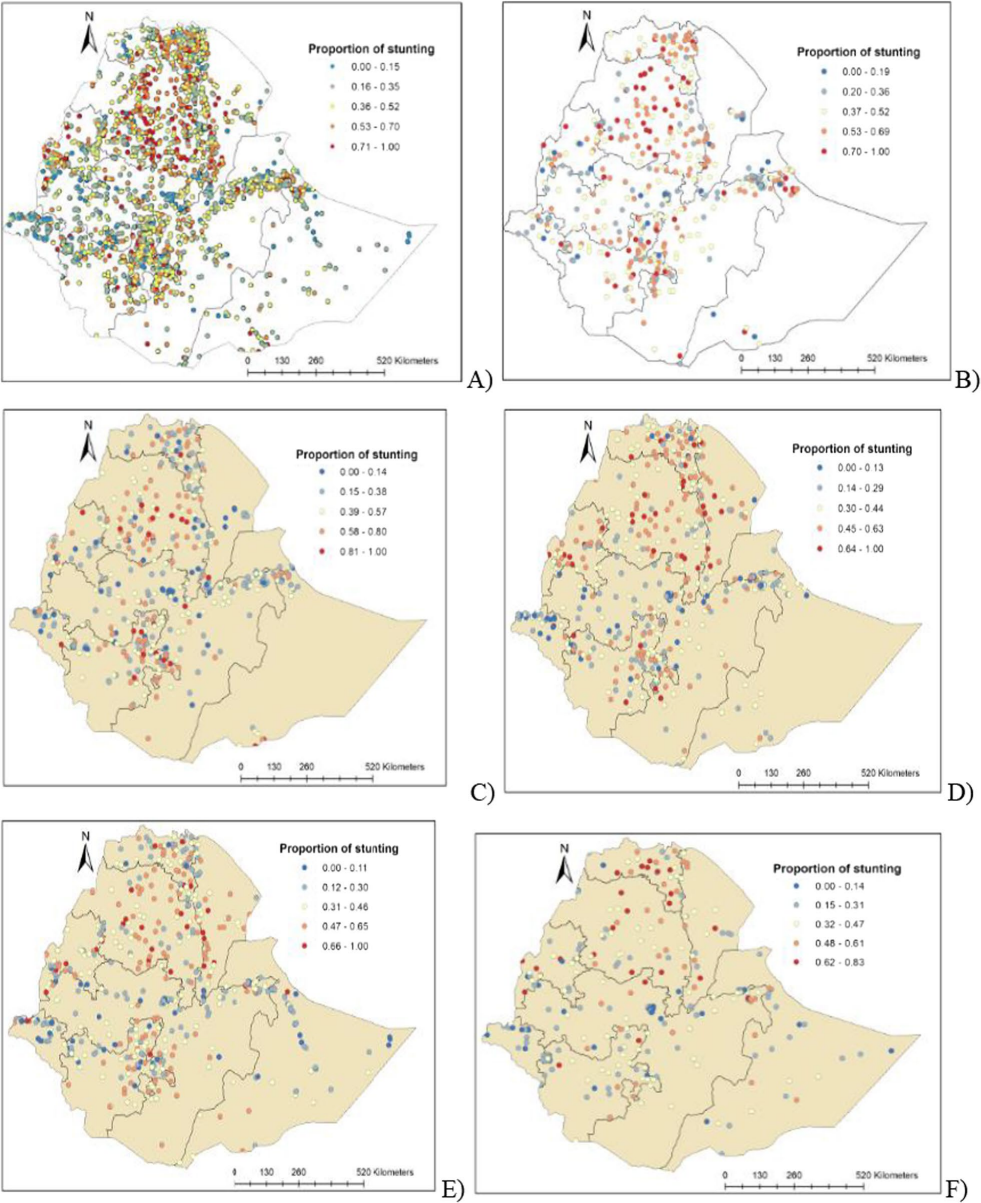
Geopspatial points and proportion of stunting in Ethiopia **A** 2000–2019 **B** 2000, **C** 2005, **D** 2011, **E** 2016, and **F** 2019

The spatial clustering of stunting was observed in the Northern, and North central parts of Ethiopia (Fig. 2A). Clustering of stunted children was observed in Northcentral parts of the country (i.e., Central and Northern Amhara and Tigray regions) in 2000 EDHS (Fig. 2B); Northcentral and Southern in 2005 EDHS (Fig. 2C); Northern in 2011 and 2019 (Fig. 2D, F); and Northern and Southern parts of the country in 2016 (Fig. 2E).

**Fig. 3:**
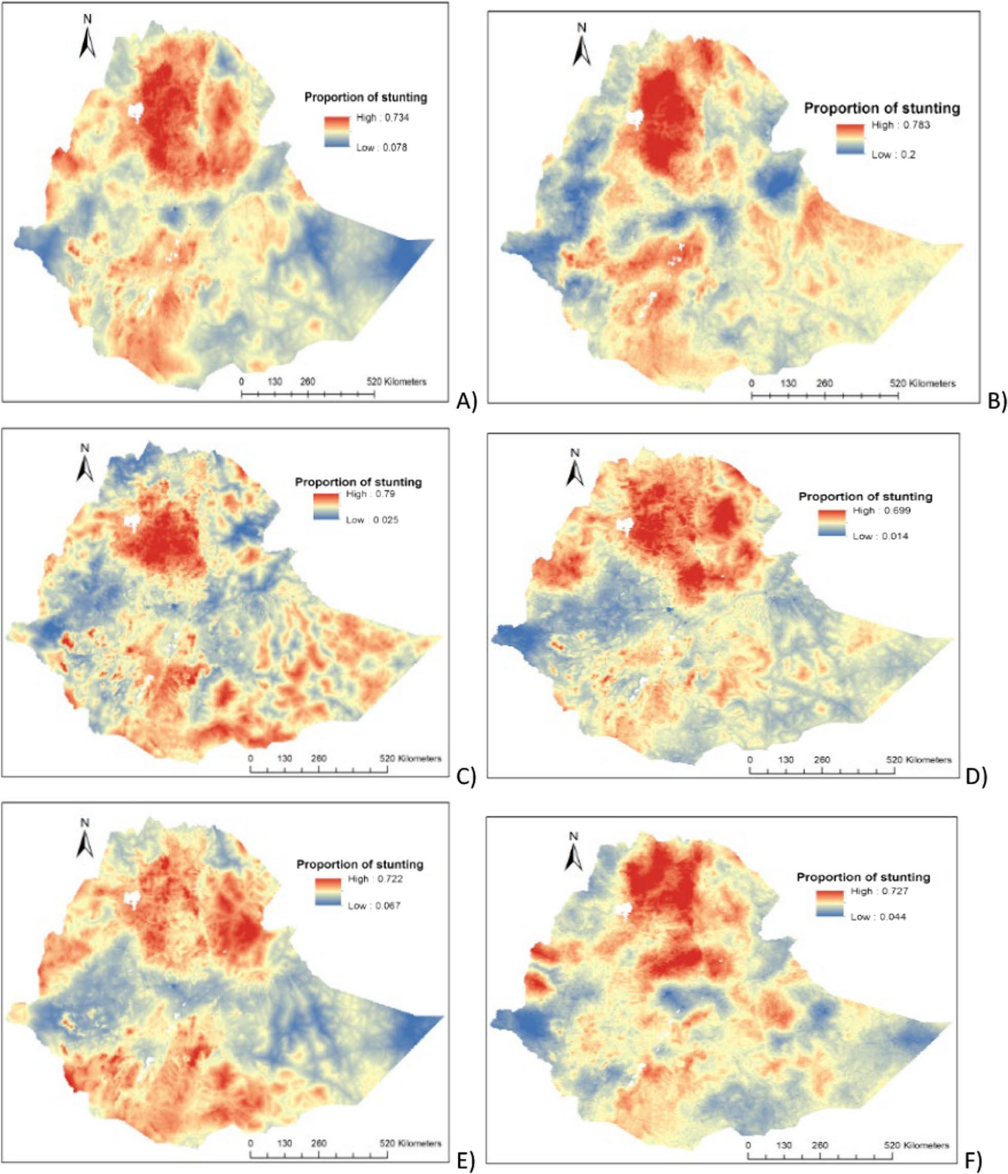
The predicted geospatial map the proportion of stunting among under-five children in Ethiopia: **A** 2000–2019, **B** 2000, **C** 2005, **D** 2011, **E** 2016 and **F** 2019

The highest predicted prevalence of stunting was observed in the Northern, Northcentral, Northwestern, and Southern parts of the country (Fig. 3A–F).

### Regression analysis

We used the Bayesian geostatistical model to identify drivers of stunting among under-five children in Ethiopia. Temperature (mean regression coefficient (β): −0.19; 95% credible interval (95% CrI): −0.25, −0.12) and population density (β: −0.012; 95% CrI: −0.016, −0.009) were negatively associated with stunting, whereas travel time to the nearest cities (β: 0.12; 95% CrI: 0.064, 0.17) was positively associated with stunting in Ethiopia (Table 2).

**Table 2:** Regression coefficient mean and 95% credible intervals (CrI) of covariates included in a Bayesian spatial model with Binomial response for the prevalence of stunting in Ethiopia between 2000 and 2019

Covariates	Mean regression coefficients of stunting prevalence with 95% credible interval in each year					
	2000–2019	2000	2005	2011	2016	2019
Intercept	−0.45 (−0.64, −0.27)	−0.04 (−0.27, 0.19)	−0.12 (−0.48, 0.26)	−0.47 (−0.72, −0.25)	−0.46 (−0.84, −0.11)	−0.58 (−0.81, −0.37)
Temperature	**−0.19 (−0.25, −0.12)**	**−0.14 (−0.24, −0.03)**	**−0.23 (−0.37, −0.08)**	**−0.11 (−0.21, −0.01)**	**−0.19 (−0.31, −0.08)**	**−0.18 (−0.34, −0.02)**
Precipitation	0.017 (−0.09, 0.13)	−0.05 (−0.20, 0.09)	0.001 (−0.21, 0.23)	0.02 (−0.14, 0.18)	−0.02 (−0.21, 0.18)	−0.04 (−0.23, 0.17)
Travel time to cities	**0.12 (0.06, 0.17)**	**0.11 (0.001, 0.22)**	**0.17 (0.00, 0.35)**	**0.14 (0.05, 0.24)**	**0.17 (0.05, 0.29)**	0.11 (−0.06, 0.28)
Population density	**−0.012 (−0.02, −0.01)**	**−0.01 (−0.01, −0.001)**	**−0.02 (−0.04, −0.01)**	**−0.03 (−0.04, −0.02)**	**−0.01 (−0.02, −0.01)**	**−0.01 (−0.02, −0.001)**
Distance to water	−0.014 (−0.04, 0.01)	−0.04 (−0.09, 0.02)	0.001 (−0.07, 0.08)	0.02 (−0.03, 0.07)	0.01 (−0.05, 0.06)	−0.05 (−0.14, 0.03)
Healthcare access	0.009 (−0.07, 0.09)	−0.01 (−0.23, 0.22)	0.15 (−0.11, 0.41)	−0.03 (−0.18, 0.11)	0.07 (−0.10, 0.23)	−0.03 (−0.24, 0.17)

N.B. The bold text in the table indicates the statistical significance

Widely Applicable Information Criteria (WAIC) statistics were used to identify the best-fitted model and the model with the lowest WAIC value was the best-fitted model.

## Discussion

This study found that the national prevalence of stunting in under-five children varies at subnational and local levels. The spatial distributions of stunting were also varied temporally. The overall national prevalence of stunting in Ethiopia varies from 47.9% in 2000 to 35.9% in 2019, which indicates a 25% reduction in the past twenty years. The reduction in the prevalence of stunting is due to the implementation and expansion of a community health extension program by the Ethiopian government since 2004, which aimed to promote disease prevention and control including screening, treatment, and counseling for malnutrition.

The overall prevalence of stunting among under-five children in Ethiopia is far below the 2025 global target, which states a 40% reduction of stunting in under-five children through a comprehensive implementation of maternal, infant, and young children. This could be because of high food insecurity, poor socioeconomic status, and lack of adequate knowledge about child nutrition, particularly in the rural parts of Ethiopia [30, 31].

In line with previous studies conducted in Ethiopia [9, 32], the spatial clustering of stunting was observed in the Northern, Northcentral and Northwestern parts of the country. The reason could be linked with child feeding practices, crop production, and the fertility of the farmland [33].

The spatial binomial modeling was fitted using the Bayesian framework to identify drivers of stunting. Hence, the temperature is negatively associated with stunting among under-five children, which is supported by previous studies conducted in India [34], Bolivia [35], Argentina [36], United States of America [37], Peru [38], Tanzania [39], and Ethiopia [40]. Even though the scientific evidence was limited to explaining the relationship between temperature and stunting [40, 41]. In Ethiopia, agricultural activities decrease in areas with high temperatures, which leads to chronic starvations and stunting. Altered climate such as increased temperature has an impact on the availability, access, and usage of foods [42, 43]. Previous studies were also supported the effect of high temperature on stunting [44, 45]. In contrast with previous studies chronic hypoxia is common on the highlands, which have a deleterious impact on various phenotypic traits that might impact the physical growth [37].

Furthermore, the population density was negatively associated with stunting, which is in agreement with a study done in Yemen [46] and trend analysis of urban areas and developing nations reports [47]. Population density is higher in urban areas, where there is better awareness of child nutrition, a higher wealth index, and better healthcare access. On the other hand, population density is lower in rural, where poor sanitation, inadequate knowledge of child nutrition, and poor socioeconomic status are highly prevalent [39, 48].

Moreover, travel time to the nearest cities in minutes was positively associated with stunting among under-five children, which means when the travel time to the cities increases the prevalence of stunting is increased. This might be related to healthcare access and improved child nutrition, poor sanitation, and infrastructure [49, 50].

This study has paramount importance for policymakers and program designers to come up with evidence-based interventions in reducing the burden of stunting, to meet the WHO 2030 targets. Understanding the trend and distribution will help to design timely area-specific interventions. Identifying the drivers of stunting over time could help to generate evidence targeted to each specific driver.

The strength of this study was using nationally representative data, which could produce reliable estimates with advanced geostatistical analysis. However, this study had some limitations, the use of covariates is limited to open sources online data, which limits the number of covariates. In addition, some important clinical covariates were not included in this analysis.

## Conclusion

The prevalence of stunting varied substantially at subnational and local levels over time. Spatial clustering of stunting was observed in Northern Ethiopia. Temperature, population density and travel time to the nearest cities were identified as the drivers of stunting among under-five children in Ethiopia. Thus, community health extension programs should be strengthened through community awareness creation towards child nutrition, particularly in areas far from the cities.

## Data Availability

All data relevant to the study are included in the article or uploaded as supplementary information.
